# Cheminformatics analysis of molecular datasets of transcription factors associated with quorum sensing in *Pseudomonas aeruginosa*†

**DOI:** 10.1039/d1ra08352j

**Published:** 2022-02-28

**Authors:** Felipe Victoria-Muñoz, Norberto Sánchez-Cruz, José L. Medina-Franco, Fabian Lopez-Vallejo

**Affiliations:** Universidad Nacional de Colombia, Sede Bogotá, Facultad de Ciencias, Departamento de Farmacia Av. Cra 30 # 45-03, Bogotá D.C. 11001 Colombia; Universidad Nacional de Colombia, Sede Bogotá, Facultad de Ciencias, Departamento de Química, Grupo de Investigación en Productos Naturales Vegetales Bioactivos Av. Cra 30 # 45-03, Bogotá D.C. 11001 Colombia fhlopezv@unal.edu.co; DIFACQUIM Research Group, Department of Pharmacy, School of Chemistry, Universidad Nacional Autónoma de México, Avenida Universidad 3000 Mexico City 04510 Mexico

## Abstract

Transcription factors associated with quorum sensing in *P. aeruginosa* are promising targets for discovering new adjuvants against infection with this pathogen. Regulation of these transcription factors offers the possibility of controlling multiple virulence factors related to them as biofilm development, proteases, hydrogen cyanide, among others. Numerous molecules have been tested against these targets, however, the keys responsible for antagonistic activity are still unknown. In this work, the structure–activity relationships of active molecules tested against LasR, PqsR, and RhlR transcription factors are analyzed in order to establish the structural characteristics associated. As part of the study, molecular complexity, scaffold, activity cliffs, and chemical space visualization analyses were conducted to find out characteristics associated with biological activity. In this study, several structural features were identified as significant for antagonist activity, highlighting molecular size and hydrogen bond acceptors.

## Abstract 2

There are molecular structural features that are key to defining the agonist or antagonist activity on LasR, RhlR and PqsR transcription factors, associated with quorum sensing in *Pseudomonas aeruginosa*.

## Graphical abstract

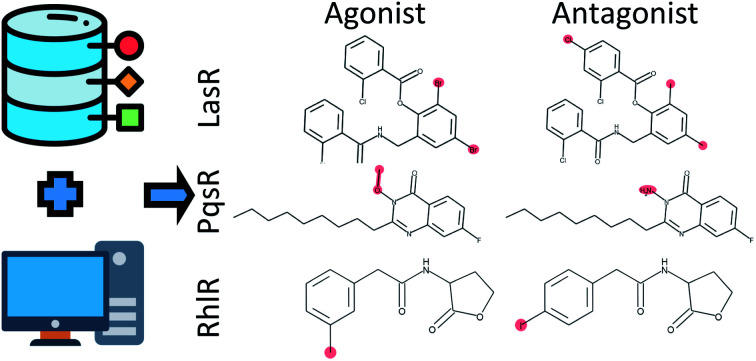

## 1 Introduction

Infection with *P. aeruginosa* is a challenge in therapeutic management. These bacteria can adapt to a hostile environment, resist the immune system, and block the action of broad-spectrum antibiotics such as third-generation cephalosporin, carbapenems, and others.^1,2^ For this reason, the World Health Organization has classified *P. aeruginosa* as a critical priority for the discovery of new drugs.^3^

One of the critical mechanisms related to the augmented antibiotic resistance of *P. aeruginosa* is quorum sensing (QS) which works like a communication system where each bacteria can detect an existent population in a given environment.^4^ Small molecules called autoinducers (AIs) regulate the QS through their binding to specific receptors. They activate the genetic machinery to produce multiple virulence factors.^5^

At present, twenty transcription factors (TFs) involved in QS are known; all of them keep multiple interactions with each other.^6^ Las, Rhl, PQS, and IQS are related to the activation of QS and the production of multiple virulence factors (elastase, protease, rhamnolipids, pyocyanin, hydrogen cyanide);^7,8^ Las and Rhl are systems of the Lux type, while PQS is a quinolone dependent system.

**Fig. 1:**
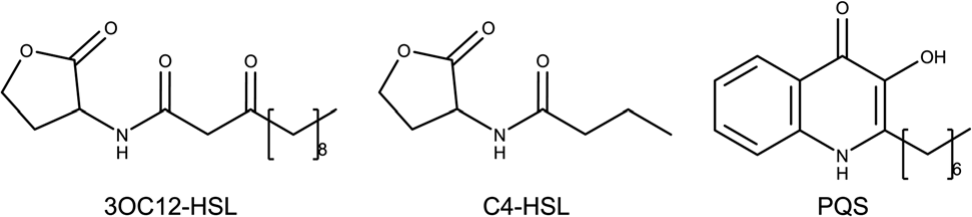
Molecular structures of autoinducers associated with quorum sensing in *P. aeruginosa*. *N*-3-Oxo-dodecanoyl homoserine lactone (3OC12-HSL), butyryl homoserine lactone (C4-HSL) and 2-heptyl-3-hydroxy-4-quinolone (PQS).^4^

Las and Rhl systems are key to QS activation. These systems have an inductor protein (LasI and RhlI) and a receptor protein (LasR and RhlR); inductors synthesize AIs and receptors recognize them. Lux type systems work with acyl-homoserine lactone molecules like AIs. These molecules are composed of a lactonic ring and a carbonate chain of variable size connected by an amide. *N*-3-Oxo-dodecanoyl homoserine lactone (3OC12-HSL) is the autoinducer of the Las system, while butyryl homoserine lactone (C4-HSL) is an autoinducer of the Rhl system (Fig. 1).^4,9^

2-Heptyl-3-hydroxy-4-quinolone (PQS) is the associated autoinducer to PQS system. PQS is recognized by PqsR and synthesized by multiple proteins such as PqsB, PqsC, PqsD, among others.^10–12^ 2-(2-Hydroxyphenyl)-thiazole-4-carbaldehyde (IQS) is an autoinducer for IQS system. Previous studies established the activation of QS by IQS in deficiency of Las system, generating interest for their results.^7,13^ LasR, RhlR, and PqsR AIs are shown in Fig. 1.

Detecting new antagonists for these TFs gives innovative pathways for the *P. aeruginosa* infection management, nevertheless the structure–activity relationship of these targets is not known yet, highlighting interesting computational studies conducted on this topic.^14–16^ For this reason, this paper proposes some relevant keys to contribute to solving this problem by searching for characteristics or structural patterns associated with agonist, antagonist activity, or inactivity. To achieve this goal, a dataset was built of 289 molecules with reported biological activity in major public repositories from LasR, PqsR, and RhlR, and cheminformatics studies were performed.

## 2 Methods

### 2.1 Construction and preparation of compound datasets

Compound datasets (DS) were built with ChEMBL version 26, PubChem, and scientific articles published in peer-reviewed journals. For ChEMBL, the following identifiers were used: LasR – CHEMBL1075207, PqsR – CHEMBL2424500, and RhlR – CHEMBL3112386. In PubChem bioassay, the search was done directly with AIs names. Additionally, for the previous search, only molecules with IC_50_ and EC_50_ values were taken into account. Keywords ‘LasR’, ‘PqsR’, ‘RhlR’ and ‘quorum sensing’ were used to search for molecules in scientific articles at SciFinder. Afterwards, all the results were filtered taking into account their biological activity values ‘IC_50_’ or ‘EC_50_’ according to all the articles published between 1998 and 2020.

All the molecules in DS were classified as “active” or “inactive” using a cutoff value of 10 μM for LasR and PqsR, while 12 μM was used for RhlR. These cutoff values were chosen based on the mean minus one standard deviation of biological activity. For a better understanding, some analysis required the pIC_50_ calculation (−log IC_50_). Then, the following steps were applied to prepare the molecules: (1) the largest fragment in each molecule was selected; (2) non-organic molecules were removed; (3) charges were neutralized by adding and/or removing hydrogens where possible; (4) it was ensured that the most potent acid groups were first ionized in partially ionized molecules; (5) stereochemistry was removed; (6) the most populated tautomer was selected; (7) duplicate molecules were discarded. This curation was done with RDKit and MolVS libraries for Python.^17,18^

### 2.2 Molecular complexity

Molecular complexity has been associated with target selectivity and success in clinical stages.^19–21^ Here the relation between active compounds and molecular complexity was examined. For this study, complexity metrics based on topological descriptors like the fractions of sp^3^ atoms (Fsp^3^) and chiral centers (FCC) were used.^22^ Likewise, the Bertz and DataWarrior complexity were calculated.^23,24^ All the metrics were computed with RDKit and DataWarrior 5.0.0.^17,24^

### 2.3 Scaffolds analysis

Scaffolds can be defined as the core of a molecule used in drug design.^25–27^ Scaffolds are intuitive and informative when exploring SAR and Structure-Multiple Activity Relationships (SmART) of compounds datasets as a way of identifying supports to drug design.^28^ This work explores the relationships between scaffolds and the biological activity for LasR, PqsR, and RhlR datasets. For scaffold generation, in accordance with Bemis and Murcko as implemented in RDKit,^17,29^ a scaffold is defined as the core structure of a compound consisting of all of its rings and connecting linkers. A maximum common substructure analysis was done on the most frequent scaffold in each DS.^30,31^ The data were collected using RDKit.^17^

### 2.4 Activity cliffs analysis

Activity cliffs are small changes in the structure associated with unexpected large changes in biological activity.^32–34^ Identification of activity cliffs is a relevant and informative analysis of the SAR because they point to specific and minor structural modifications that strongly influence biological activity. It is important to note, however, that there might be “artificial” cliffs (or artifacts) in compound data sets, for instance, when the biological activity is not measured correctly.^35^ In this work, to identify the possible presence of activity cliffs in the DS, consensus activity cliffs were calculated between multiple 2D fingerprints (MACCS Keys 166-bits, ECFP4, ECFP3, ECFP2 and path length) as is proposed by Medina-Franco and coworkers,^36^ finding the activity cliff common in all the fingerprints with Tanimoto coefficient. RDKit and MayaChemTools^44^ were employed for this analysis.^17,37^

### 2.5 Chemical space visualization

The representation of a set of molecules in a two- or three-dimensional plot based on a set of pre-defined descriptors is usually known as visualization of the chemical space. In this work, the statistical analysis called t-distributed Stochastic Neighbor Embedding (t-SNE) was done so as to represent chemical space occupied by datasets, with a perplexity equal to 10, a learning rate equal to 100, using the Jaccard index as distance metric and ECFP4 to represent the compounds. Additionally, constellation plots recently developed were built for the datasets.^38^ These plots integrate a methodology of analog series with the visualization of the chemical space, associating the molecular analog to several molecules and their biological activity.

## 3 Results and discussion

Table 1 summarizes the unique compounds found in ChEMBL, PubChem Bioassays and the scientific papers. In more detail, for ChEMBL can be found molecules with LasR, PqsR, and RhlR activities. For instance, for LasR there are 173 molecules reported, but only 82 have IC_50_ and EC_50_ values; 29 molecules are reported for PqsR, of which 8 have EC_50_ values; while from 31 compounds found for RhlR, only 4 of them have IC_50_ values. In comparison, PubChem Bioassay has 87, 10, and 17 compounds with activity for LasR, PqsR, and RhlR, respectively. The remainder of the molecules was found in scientific articles. More than 70 percent of molecules are shared with ChEMBL. LasR is the TF with the highest number of molecules. Furthermore, the LasR dataset has the highest proportion of active molecules among the three datasets. These datasets can be found in the ESI.†

**Table 1:** TFs datasets with unique compounds

DS	Total	Agonist		Antagonist		Inactive	
		*n*	%	*n*	%	*n*	%
LasR	188	52	27.7	41	21.8	95	50.5
PqsR	54	10	18.5	15	27.8	29	53.7
RhlR	47	12	25.6	7	14.9	28	59.5

### 3.1 Molecular complexity

**Fig. 2:**
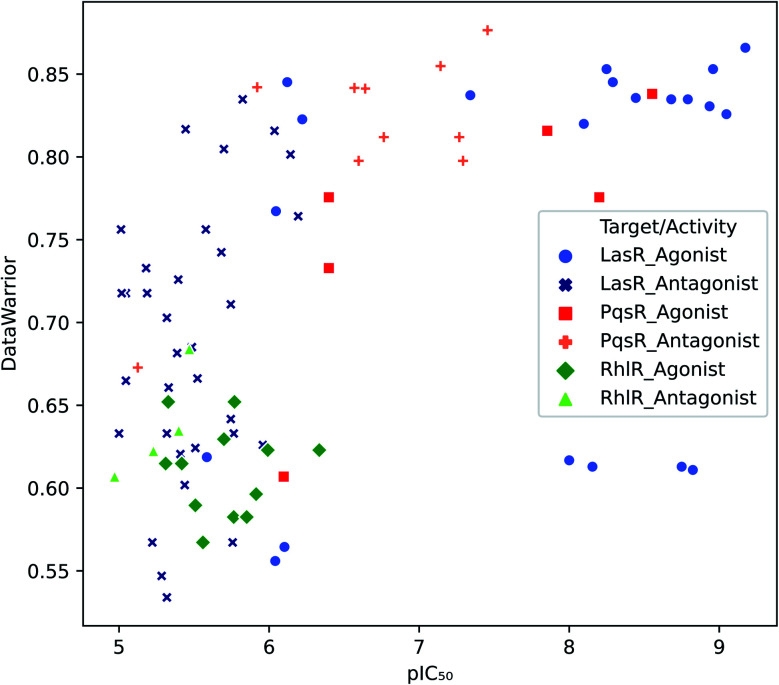
Distribution of biological activity values against DataWarrior complexity. Molecules with activity against LasR, PqsR and RhlR are in blue, red and green respectively.

Target selectivity is a major issue in drug discovery. The relationship between higher molecular complexity and less target promiscuity had already been suggested.^19,20,22,39,40^ In the present study, an association between DataWarrior complexity and biological activity was found as shown in Fig. 2.

The majority of PqsR active molecules have the highest DataWarrior complexity and some of them have pIC_50_ higher than 7. In contrast, RhlR compounds are towards the left bottom corner of Fig. 2, where molecules with low activity and poor complexity are located. Finally, LasR active compounds could not be associated with specific complexity values because this dataset has a high molecular diversity as it is evidenced in analysis further on. Antagonist molecules in all the datasets do not have a clear behaviour associated with complexity for the metrics studied. These results are available in the ESI.†

### 3.2 Scaffold analysis

**Fig. 3:**
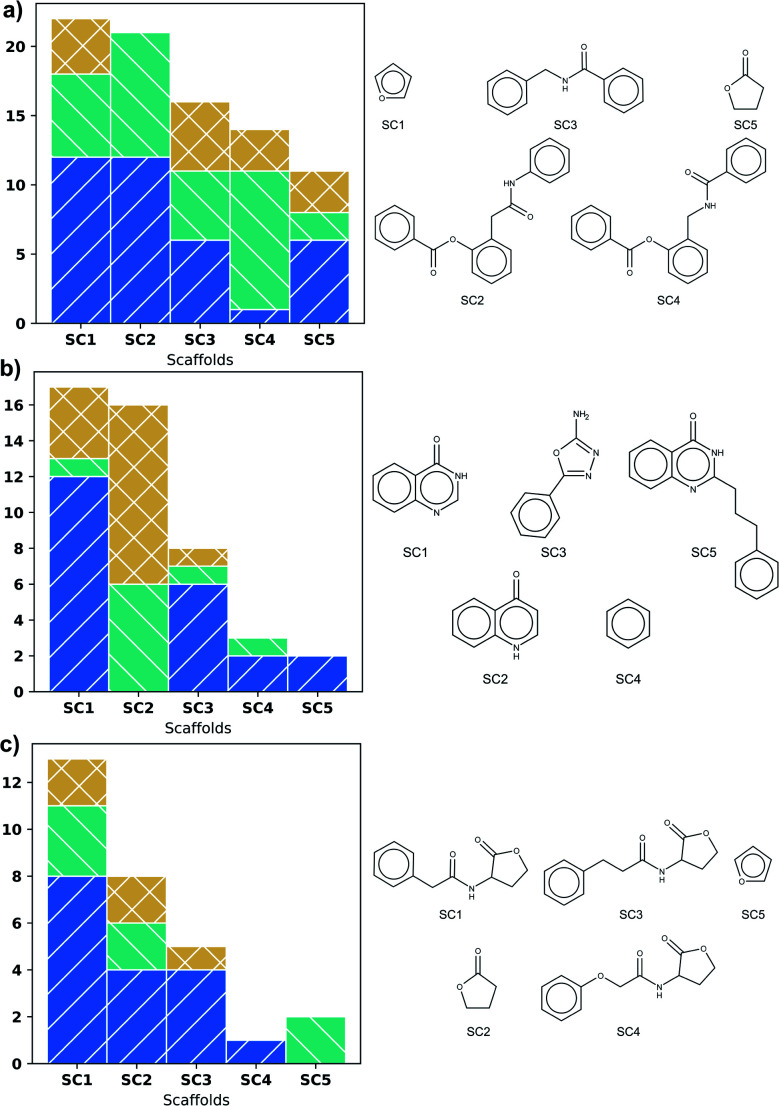
First five most frequent Bemis-Murcko scaffolds in: (a) LasR dataset, (b) PqsR dataset and (c) RhlR dataset. Activity type is represented by colours: inactive (blue), agonist (green), and antagonist molecules (orange).

Fig. 3 shows scaffold frequency of the DS on the left side; scaffold structures are displayed on the right. The frequency of scaffolds found for DS varies according to DS size (Fig. 3). Nevertheless, the five most frequent scaffolds represent around 20% of the total, highlighting a variety of types of biological activity in these scaffolds. In LasR, it is found that those first five scaffolds have molecules with activity, being the most frequent the agonist activity, which is shown in Fig. 3a. Rings of six members connected by a short amide chain are the most relevant ones for this DS.

Three phenyl system is present in the second and fourth most populated scaffolds in Fig. 3a but the position of amide in chain allows antagonistic activity for the fourth scaffold. Even though SC1 and SC2 scaffolds are based on the common lactone ring in autoinducers, they show all the three activities, therefore these scaffolds are unsuitable for similarity search. SC3 and SC4 could be useful.

Scaffolds of PqsR DS are compounds with six-membered rings fused, a common feature of the natural autoinducer. All the molecules with quinoline ring (SC2) have an activity lower than 10 μM (Fig. 3b), making it a suitable candidate for similarity search of new molecules with potential biological activity. It is important to mention that the first two scaffolds (SC1 and SC2) represent more than fifty percent of the molecules, revealing a low scaffold diversity in this DS.

The biological activity of the first five scaffolds found in RhlR DS is higher than the activity of scaffolds in the rest of DS. Here all the scaffolds have a lactone ring, but scaffold (SC4), the one with the largest percent of antagonist molecules, also presents an amide chain with an ether and a phenyl moiety.

**Fig. 4:**
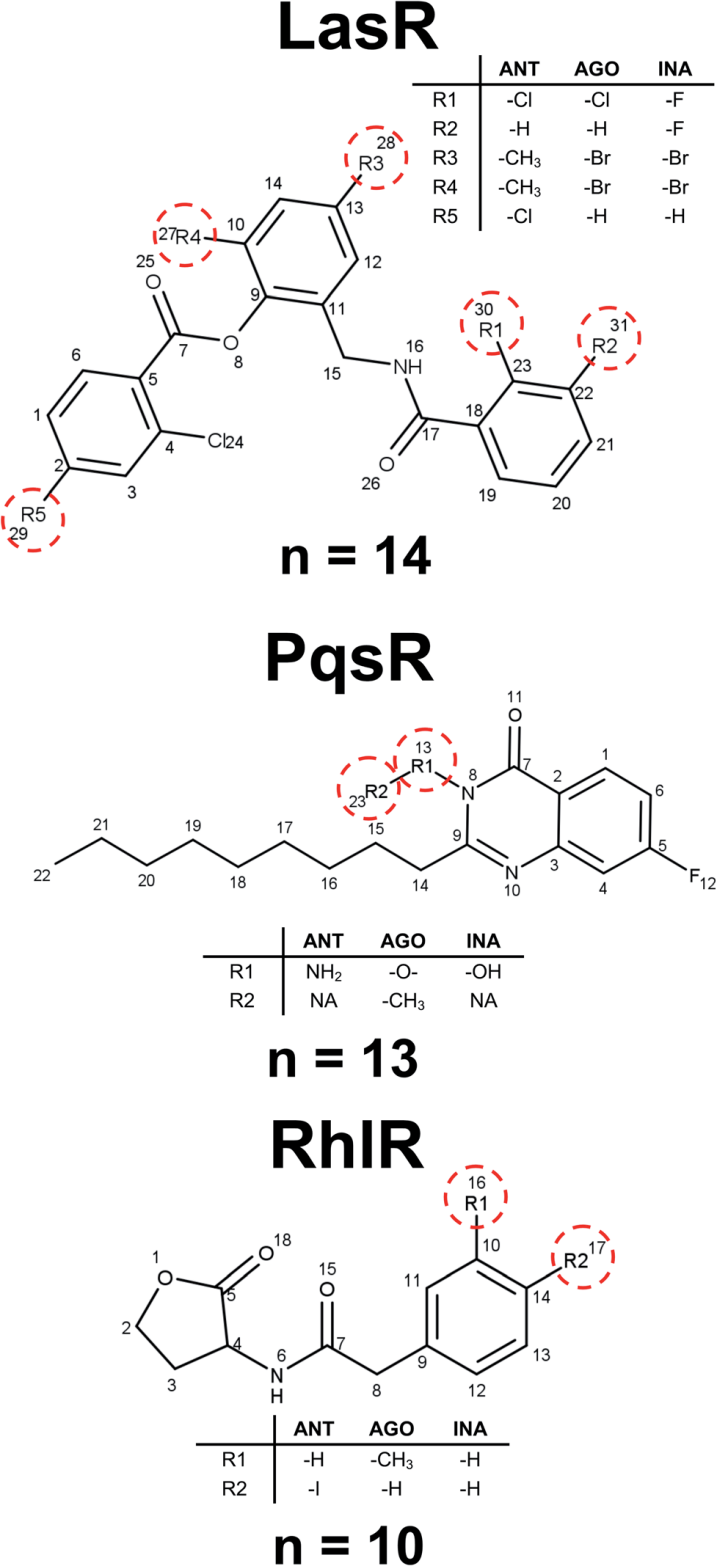
Representative examples of maximum common substructures. *n* represents the number of molecules with this scaffold.

Analysis of the maximum common substructure (MCS) allows to detect small structural differences between molecules with different types of activity. All the molecules shown in Fig. 4 for LasR DS were reported by O'Reilly and collaborators,^41^ they have a triphenyl scaffold showing the activity related to a specific ring substitution. The inactive molecule is *ortho* and *meta* disubstituted by fluorine atom in the first ring, the agonist and antagonist are monosubstituted in the *ortho* position by chlorine atom in the first ring, but the antagonist is additionally monosubstituted in the *para* position in the third ring. These substitutions can change the inductive effect generating in this way different interactions in the binding site.^42^

Ilangovan *et al.* reported several compounds associated with PqsR activity.^43^ Those compounds differ from each other only in the substitution of nitrogen in the quinazoline ring: the inactive compound has a hydroxyl group, the agonist has ethyl, and the antagonist has an amine, finding the association of biological activity related to hydrogen bonds acceptors and donors of this position (Fig. 4). For RhlR DS, it is possible to establish a steric effect in the binding site with the type of activity, as it is shown in Fig. 4. This analysis allows to establish a mechanism of action cliffs (MOA-cliffs) for these scaffolds.^44^ All of these scaffolds and its frequency are available in the ESI.†

### 3.3 Activity cliffs

**Fig. 5:**
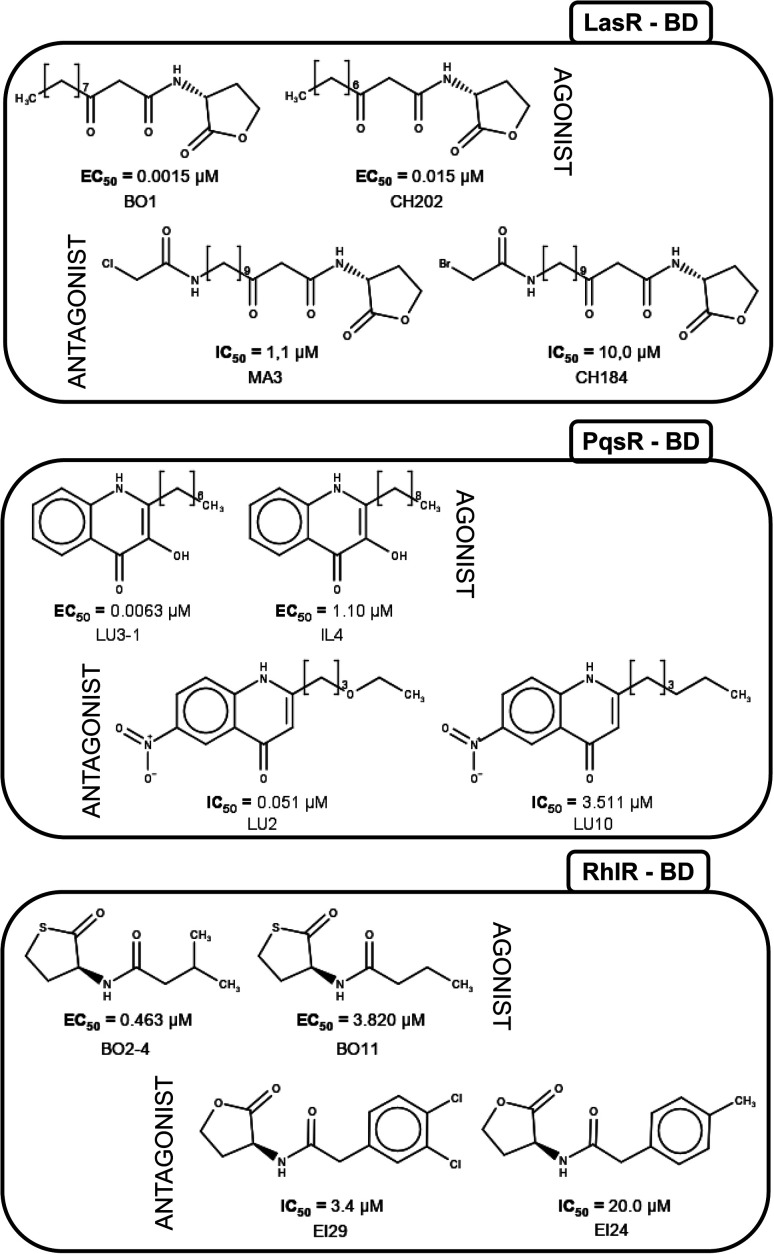
Activity cliffs found in datasets. The agonist is shown to the left and the antagonist to the right.

Fig. 5 shows the structure of representative activity cliffs of the agonists and antagonist for all the TFs. It is evident the relationship of agonist and antagonist molecules, supported by our previous analysis. Regarding LasR agonists, a change of the EC_50_ values can be noted by a variation in one carbon on the side chain: BO1 has a side chain of 11 carbons, while CH202 has 10 carbons, causing a 1-fold decrease in biological activity. Furthermore, natural autoinducer has a 12-carbons side-chain. All this allows us to understand the importance of the size of side-chain in agonist activity. The molecular pair found for the antagonist activity of LasR, MA3 and CH184, differ in the halogen atom at the end of the side-chain: MA3 has chlorine while CH184 has bromine. Thus, MA3 is about 10 times more active than CH184 relating the possibility to make a halogen bond, which could be a possible cause of antagonist activity.

LU3-1 and IL4 is the molecular pair found for agonist activity of PqsR, LU3-1 has a 7-carbon side-chain, while IL4 has a 9-carbon side-chain, agonist activity of LU3-1 is about two orders of magnitude greater than IL4, once again confirming the importance of side-chain length in agonist activity. On the other hand, the pair of compounds LU2 and LU10 were activity cliffs related to antagonist activity; both have a 6-carbons side-chain, but LU2 has an oxygen atom at position four, while in LU10 they all are carbons. This change is associated with a potency difference in two orders of magnitude. The potency difference could be explained by the lack of a hydrogen bond acceptor in LU10.

Activity cliffs for RhlR agonists are molecules with a thiolactone ring and a side-chain, here BO11 side chain changes a hydrogen atom in the fourth position to a methyl group in compound BO2–4; activity for BO2–4 is about eight times greater than BO11.

**Fig. 6:**
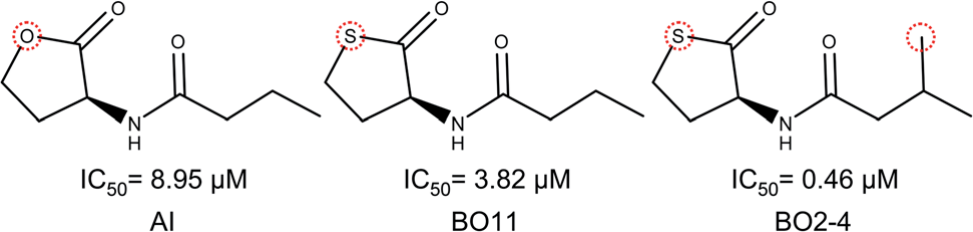
Structural comparison of autoinducer (AI) and agonist activity cliffs of RhlR dataset (BO11 and BO2–4).^40^

It is notable that the natural autoinducer of RhlR has an EC_50_ (8.95 μM) lower than these compounds, and its difference is a lactonic ring *versus* thiolactone ring as reported by Boursier and co-workers (Fig. 6).^45^ The substitution on the phenyl ring in molecular pairs found for antagonist activity generates a change of this activity, while EI29 is two chlorine-substituted, EI24 is monomethyl substituted, linking the size and inductive effects with antagonist activity.^46^

### 3.4 Chemical space

**Fig. 7:**
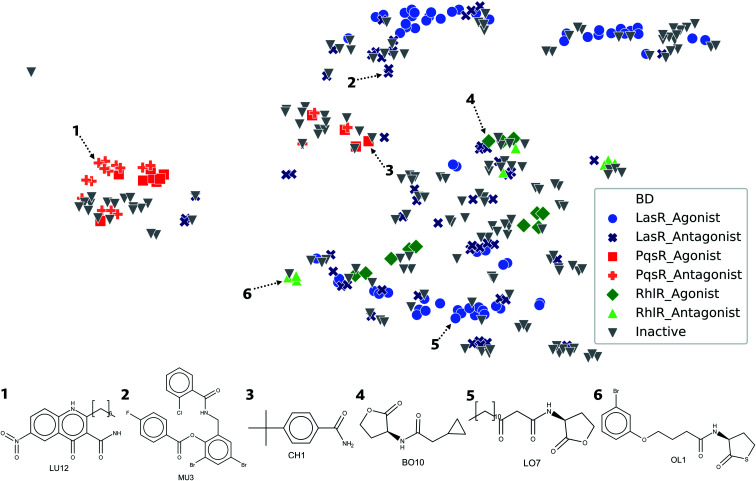
Visual representation of the structural chemical space obtained by t-distributed stochastic neighbor embedding (t-SNE). Molecules are classified according to their activity: inactive (grey), LasR agonist (light blue), LasR antagonist (dark blue), PqsR agonist (red), PqsR antagonist (pink), RhlR agonist (light green), and RhlR antagonist (dark green).

Visualization of the Euclidean distance between molecules by using structural parameters as a classification method provides a comprehensive view of the molecular data sets. Therefore, a representation was done of a chemical space of DS related to a type of biological activity, which generates a general overview of the structural diversity of the molecules (Fig. 7). The chemical space visualization, occupied by compounds in DS and based on ECFP4 fingerprints, shows that the agonist and antagonist molecules are close in space due to the fact that the type of compounds are similar to each other, according to our previous results. In the lower right corner of the plot, molecules with five-membered rings are displayed, usually lactone and thiolactone rings. In this region of the chemical space, compounds of LasR and RhlR DS can be found. LasR agonists and antagonists are closer to each other than molecules of RhlR since the spatial location is given by the structure.

In the upper right corner of Fig. 7, molecules with triphenyl scaffold are found, a common characteristic of LasR DS. However, in order to find activity cliffs, a group of antagonist molecules is displayed separated from the agonist compounds; this might be a critical starting point for optimizing the searching of antagonist activity or similarity in big databases. Compounds of PqsR DS are displayed in the center of the plot; quinoline and quinazoline rings are usual scaffolds with PqsR activity.

In the visualization of the chemical space in Fig. 7, all the DS are close in the space, especially LasR and RhlR DS. This is an indication that it is possible to find a molecule with antagonist activity against both TFs; this is attributable to its high similarity. The proximity between agonist and antagonist compounds shows the challenge of finding specific structural characteristics associated with antagonist activity against all the TFs.

### 3.5 Constellation plots

A constellation plot is a hybrid approach of visualization of chemical space and substructure analysis. As described in detail elsewhere,^38^ the main analog series of a compound data set were generated. The structural similarity of the analog series is displayed in a two-dimensional representation of chemical space such as t-SNE. Constellation plots represent a method to visualize and analyse StARs (Structure–Activity Relationships) in chemical space.^47,48^

**Fig. 8:**
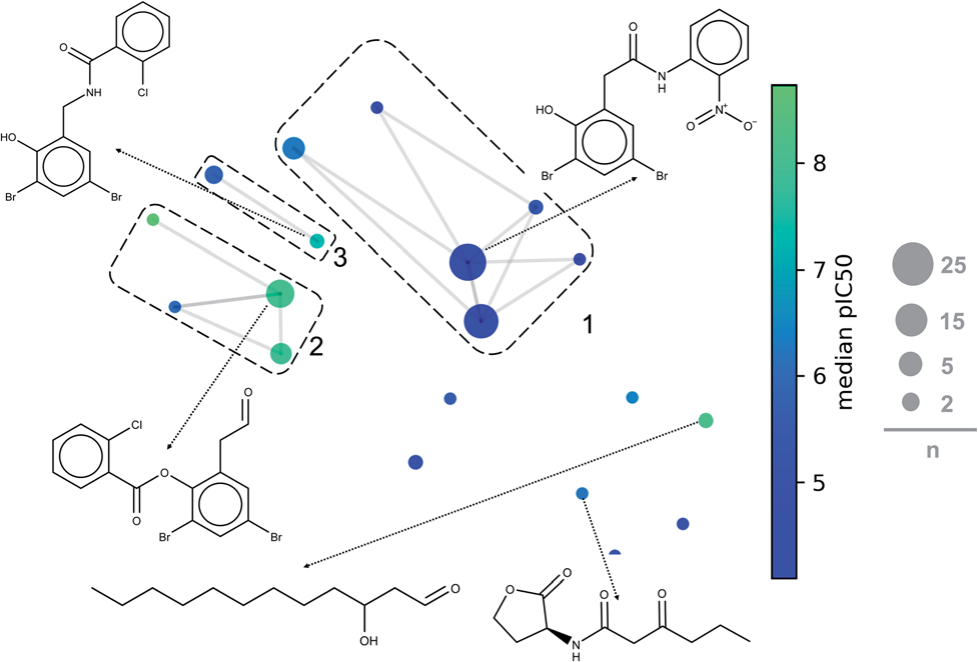
General constellation plot for a dataset of LasR. The most representative cores are shown. This plot has the same parameters of Fig. 7 (t-SNE, ECFP4, Tanimoto Matrix). Each point represents a core, the size of circles indicates the number “*n*” of compounds annotated; connected circles are cores sharing compounds; grids represent analog series, and colour shows the average of pIC_50_.

For this paper, constellation plots were built following the methodology proposed by Naveja and Medina-Franco.^38^ These analyses provide with the relation of standard cores in the DS with biological activity and the number of compounds that they have. Constellation plot for LasR DS (Fig. 8) shows nineteen cores grouped in ten analog series; they contained one hundred and six compounds (around half of the dataset).

The first three analog series display small structural changes in diphenyl rings, both the first and third series show diphenyl rings connected by a chain of three members with an amide group in the middle of them. The second series has diphenyl rings connected by a chain of two members with an ester group in the middle of them, that series has a better activity profile which sets an excellent base for searching other compounds or a useful scaffold for optimizing biological activity.

Other essential cores for this activity are a carbonate chain of twelve members and a lactone ring. The carbon chain compounds have an excellent mean activity for this target, but the lactone ring does not; this is because the lactone ring has multiple compounds with a harmful activity while carbon chain has a significant number of compounds with functional activity. PqsR and RhlR DS could not be analysed with this method because the rules of fragmentation used does not involve carbon chains or rings.^43^

## 4 Conclusions

PqsR active molecules showed a high DataWarrior complexity. In RhlR DS, all the molecules were correlated with low DataWarrior complexity; in contrast, LasR active compounds were associated with both high and low complexity values, making impossible a specific association with DataWarrior complexity.

For LasR antagonist compounds, it was identified that the side-chain size in homoserine lactone scaffolds should range from 10 to 12 atoms. In contrast, the rings substitution in triphenyl scaffolds generates a specific type of activity. In molecules active against PqsR, the size of the side-chain quinazoline rings and substitution on the nitrogen result in a certain type of activity. Active homoserine lactone scaffolds for RhlR show that phenyl or ethyl substitution on the side-chain does give also a type of activity.

The maximum common substructure analysis and chemical space visualization have indicated that there is no scaffold associated with a unique type of activity in any TF. Yet, the fact that most of them are active should be considered as an encouraging compound to optimize. The LasR constellation plot allowed to determine that the diphenyl scaffold is one of the most promising for optimizing molecules since it is present in more than 25 molecules with LasR biological activity.

Nevertheless, it is important to highlight the ambiguity of these characteristics as in some cases it could generate a change in the mode of action, as described in MOA-cliffs, making compound optimization difficult. To establish the structural keys with a type of activity, it is advisable to use molecular docking and dynamics simulations for a deeper understanding at the molecular level.

## Author contributions

Felipe Victoria-Muñoz: conceptualization, data curation, formal analysis, methodology, writing – original draft. Norberto Sanchez-Cruz: conceptualization, formal analysis, methodology, data curation. Jose L Medina-Franco: conceptualization, methodology, formal analysis, review & editing, supervision. Fabian Lopez-Vallejo: conceptualization, methodology, formal analysis, review & editing, project administration, supervision, funding acquisition.

## Conflicts of interest

The authors declare that there is no financial or commercial conflict of interest.

## Supplementary Material

RA-012-D1RA08352J-s001
