# Supplementary material for: Cheminformatics analysis of molecular datasets of transcription factors associated with quorum sensing in Pseudomonas aeruginosa
Source: RSC Adv. 2022 Feb 28;12(11):6783–90. doi: 10.1039/d1ra08352j (PMC8981735; doi:10.1039/d1ra08352j)
Supplement: RA-012-D1RA08352J-s001 [file RA-012-D1RA08352J-s001.pdf]

Electronic Supplementary Material (ESI) for RSC Advances.
This journal is © The Royal Society of Chemistry 2022

Supplementary Information

1. Molecular datasets used
2. Complexity graphs
3. Scaffolds founded

1. Molecular datasets used
    a. LasR Dataset

Molecule	Activity	pIC_{50}	Reference
S(SCCCCCCCCCCC(=O)CC(=O)N[C@H]1CCOC1=O)(=O)(=O)C	INACTIVE	5.82	1
S=C=NC[C@H](F)CCCCCCCC(=O)CC(=O)N[C@H]1CCOC1=O	INACTIVE	3.81	1
Br[C@H](CCCCCCCC(=O)CC(=O)N[C@H]1CCOC1=O)CN=C=S	INACTIVE	4.02	1
Cl[C@H](CCCCCCCC(=O)CC(=O)N[C@H]1CCOC1=O)CN=C=S	INACTIVE	3.84	1
SCCCCCCCCCCCC(=O)CC(=O)N[C@H]1CCOC1=O	INACTIVE	3.94	1
O1CC[C@H](NC(=O)CC(=O)CCCCCCC[C@H]2OC2)C1=O	AGONIST	7.64	1
S(SCCCCCCCCC(=O)CC(=O)N[C@H]1CCOC1=O)(=O)(=O)C	INACTIVE	5	1
S(SCCCCCCCCCC(=O)CC(=O)N[C@H]1CCOC1=O)(=O)(=O)C	INACTIVE	5.22	1
O1CC[C@H](NC(=O)CC(=O)CCCCCCCC)C1=O	AGONIST	8.82	2
O1CC[C@H](OC(=O)CC(=O)CCCCCCCCNC(=O)CC(=O)CCCCCCCC)C1=O	INACTIVE	3.6	2
S1CC[C@@H](NC(=O)CC(=O)CCCCCCCC)C1=O	AGONIST	8.82	2
O=C1NCC[C@H]1NC(=O)CC(=O)CCCCCCCC	AGONIST	7.52	2
O=C1CCC[C@H]1NC(=O)CC(=O)CCCCCCCC	AGONIST	7.82	2
O=C(CCCCCCCC)CC(=O)NC1CCCC1	AGONIST	6.8	2
O1C[C@H](NC(=O)CC(=O)CCCCCCCC)CC1	AGONIST	6.04	2
O[C@H]1CCC[C@H]1NC(=O)CC(=O)CCCCCCCC	INACTIVE	5.72	2
O1CCC[C@H]1CNC(=O)CC(=O)CCCCCCCC	AGONIST	6.59	2
O1CC[C@@H](n2nnc(c2)CCCCCCCC)C1=O	INACTIVE	3.51	3
Brc1cc(Br)cc(CC(=O)Nc2ccccc2[N+](=O)[O-])c1OC(=O)c1ccccc1Cl	AGONIST	6.43	4
Brc1cc(Br)cc(CC(=O)Nc2ccccc2[N+](=O)[O-])c1OC(=O)c1ccccc1C	AGONIST	6.12	4
Brc1cc(Br)cc(CC(=O)Nc2ccccc2[N+](=O)[O-])c1OC(=O)c1cc(ccc1)C	INACTIVE	4.3	4
Brc1cc(Br)cc(CC(=O)Nc2ccccc2[N+](=O)[O-])c1OC(=O)c1ccc(cc1)C	INACTIVE	4.3	4
Brc1cc(Br)cc(CC(=O)Nc2ccccc2[N+](=O)[O-])c1OC(=O)c1ccccc1OC	AGONIST	6.61	4
Brc1cc(Br)cc(CC(=O)Nc2ccccc2[N+](=O)[O-])c1OC(=O)c1ccccc1[N+](=O)[O-]	AGONIST	6.31	4
Brc1cc(Br)cc(CC(=O)Nc2ccccc2[N+](=O)[O-])c1OC(=O)c1ccc(F)cc1Br	AGONIST	6.36	4
Brc1cc(Br)cc(CC(=O)Nc2ccccc2[N+](=O)[O-])c1OC(=O)c1c(Cl)cccc1Cl	AGONIST	6.5	4
Brc1cc(Br)cc(CC(=O)Nc2ccccc2[N+](=O)[O-])c1OCc1c(Cl)cccc1Cl	AGONIST	6.79	4
Brc1cc(Br)cc(CC(=O)Nc2ccccc2[N+](=O)[O-])c1OC(=O)c1ccccc1	INACTIVE	4.3	4
Brc1cc(Br)cc(CC(=O)Nc2ccccc2[N+](=O)[O-])c1OC(=O)c1cc(Cl)ccc1	INACTIVE	4.3	4
Brc1cc(Br)cc(CC(=O)Nc2cc([N+](=O)[O-])ccc2)c1OC(=O)c1ccccc1Cl	INACTIVE	4.3	4
Brc1cc(Br)cc(CC(=O)Nc2ccc([N+](=O)[O-])cc2)c1OC(=O)c1ccccc1Cl	INACTIVE	4.3	4
Brc1cc(Br)cc(CC(=O)Nc2cc(F)c(F)cc2[N+](=O)[O-])c1OC(=O)c1ccccc1Cl	AGONIST	6.48	4
Brc1cc(Br)cc(CC(=O)Nc2ncncc2)c1OC(=O)c1ccccc1Cl	AGONIST	6.22	4
Brc1cc(Br)cc(CC(=O)Nc2cc[nH+]cc2[N+](=O)[O-])c1OC(=O)c1ccccc1Cl	AGONIST	6.35	4
Brc1cc(Br)cc(CC(=O)N[C@H]2CCOC2=O)c1OC(=O)c1ccccc1Cl	AGONIST	9.05	4
Brc1cc(Br)cc(CC(=O)Nc2ncccc2)c1OC(=O)c1ccccc1Cl	INACTIVE	4.38	4
Brc1cc(Br)cc(CC(=O)Nc2ccccc2[N+](=O)[O-])c1OC(=O)c1ccc(Cl)cc1	INACTIVE	4.3	4
Brc1cc(Br)cc(CNC(=O)c2ncccc2)c1OC(=O)c1ccccc1Cl	INACTIVE	4.1	4
Brc1cc(Br)cc(CC(=O)Nc2cccc(F)c2F)c1OC(=O)c1ccccc1Cl	AGONIST	7.34	4
Brc1cc(Br)cc(CNC(=O)c2cccc(F)c2F)c1OC(=O)c1ccccc1Cl	INACTIVE	4.19	4
Brc1cc(Br)cc(CNC(=O)c2ncncc2)c1OC(=O)c1ccccc1Cl	INACTIVE	5.79	4
Brc1cc(Br)cc(CNC(=O)c2cccnc2)c1OC(=O)c1ccccc1Cl	AGONIST	6.31	4

Brc1cc(Br)cc(CNC(=O)c2ncccc2)c1OC(=O)CCCN1C(=O)C=CC1=O	INACTIVE	4.11	4
Brc1cc(Br)cc(CC(=O)N[C@@H]2CCOC2=O)c1OC(=O)CCCN1C(=O)C=CC1=O	INACTIVE	4	4
Brc1cc(Br)cc(CC(=O)N[C@@H]2CCOC2=O)c1OC(=O)CCN1C(=O)C=CC1=O	INACTIVE	4.62	4
Brc1cc(Br)cc(CC(=O)Nc2ccccc2[N+](=O)[O-])c1OC(=O)c1ccccc1Br	AGONIST	6.57	4
Brc1cc(Br)cc(CC(=O)Nc2ccccc2[N+](=O)[O-])c1OC(=O)c1cc(Br)ccc1	INACTIVE	4.3	4
Brc1cc(Br)cc(CC(=O)Nc2ccccc2[N+](=O)[O-])c1OC(=O)c1ccc(Br)cc1	INACTIVE	4.3	4
Brc1cc(Br)cc(CC(=O)Nc2ccccc2[N+](=O)[O-])c1OC(=O)c1ccccc1F	INACTIVE	5.89	4
Brc1cc(Br)cc(CC(=O)Nc2ccccc2[N+](=O)[O-])c1OC(=O)c1ccc(F)cc1	INACTIVE	4.3	4
Ic1ccccc1C(Oc1c(cc(Br)cc1Br)CC(=O)Nc1ccccc1[N+](=O)[O-])=O	INACTIVE	5.8	4
O1CC[C@H](NC(=O)CCCCCC)C1=O	INACTIVE	4.29	5
O1CC[C@H](NC(=O)C[C@H]2CCC=C2)C1=O	INACTIVE	4.48	5
O=C1CCCC[C@H]1NC(=O)CC(=O)CCCCCCCCC	ANTAGONIST	5	5
O=C([O-])Cc1nn(nn1)CCCCCCCCCCC	ANTAGONIST	7.52	5
S1CC[C@H](NC(=O)CCCCCCCCCCC)C1=O	ANTAGONIST	7.4	5
Brc1ccc(cc1)CCC(=O)N[C@H]1CCOC1=O	ANTAGONIST	6.47	5
BrCC(=O)NCCCCCCCCCC(=O)CC(=O)N[C@H]1CCOC1=O	INACTIVE	4	5
O1CC[C@H](NC(=O)CCCCCCC)C1=O	ANTAGONIST	5.76	5
O1CC[C@H](NC(=O)CCCCCCCCC)C1=O	ANTAGONIST	6.6	5
O1CC[C@H](NC(=O)CC(=O)CCCCC)C1=O	ANTAGONIST	6.96	5
Ic1ccc(cc1)CC(=O)N[C@H]1CCOC1=O	ANTAGONIST	5.76	5
O1CC[C@H](NC(=O)CC(=O)CCCCCCC)C1=O	AGONIST	7.82	5
O1CC[C@H](NC(=O)C[C@H](O)CCCCCCCCC)C1=O	AGONIST	7.27	5
O1CC[C@H](NC(=O)CC(=O)CCCCCCCCCCC)C1=O	AGONIST	8	5
O1CC[C@H](NC(=O)CC(=O)Cc2ccccc2)C1=O	AGONIST	6.27	5
Brc1cc(Br)cc(CC(=O)Nc2ccccc2[N+](=O)[O-])c1OC(=O)CCN1C(=O)CCC1=O	AGONIST	8.44	5
Brc1cc(Br)cc(CNC(=O)c2ccccc2[N+](=O)[O-])c1OC(=O)CCCN1C(=O)CCC1=O	AGONIST	6.92	5
Brc1cc(Br)cc(CNC(=O)c2ccccc2[N+](=O)[O-])c1OC(=O)c1ccccc1Cl	AGONIST	7.55	5
O1CC[C@H](NC(=O)CCCc2c3c([nH]c2)cccc3)C1=O	INACTIVE	4.83	6
Oc1ccccc1NC(=O)CC(=O)CCCCC	INACTIVE	4.43	6
O=C(CCCCCCCCC)CC(=O)Nc1ccccc1	ANTAGONIST	5.32	6
O1CC[C@H](NC(=O)Cc2cc([N+](=O)[O-])ccc2)C1=O	ANTAGONIST	6.29	6
S(Cc1nnn(c1)-c1ccc(cc1)C(=O)N[C@H]1CCOC1=O)c1ccccc1	ANTAGONIST	5.58	7
FC(F)(F)Oc1ccc(OCC(=O)N[C@H]2CCOC2=O)cc1	ANTAGONIST	5.33	8
O1CC[C@H](NC(=O)CCCCCCCCCCC)C1=O	AGONIST	7.48	8
S1CC[C@@H](NC(=O)CC(=O)CCCCCCCCC)C1=O	AGONIST	8.15	8
O1CC[C@H](NC(=O)CC(=O)CC\C=C\CCCCC)C1=O	AGONIST	8	8
ClCC(=O)NCCCCCCCCCC(=O)CC(=O)N[C@H]1CCOC1=O	ANTAGONIST	5.96	9
O=C([O-])Cc1nn(nn1)CCCCCCCCCCCCC	INACTIVE	4.52	9
S1CC[C@H](NC(=O)CC(=O)CCC)C1=O	INACTIVE	4.89	10
Brc1ccc(cc1)CC(=O)N[C@H]1CCSC1=O	AGONIST	6.4	10
S1CC[C@H](NC(=O)Cc2ccc(cc2)-c2ccccc2)C1=O	INACTIVE	5.54	10
S1CC[C@H](NC(=O)CCc2[nH]c3c(c2)cccc3)C1=O	ANTAGONIST	5.74	10
S1CC[C@H](NC(=O)CCCCC)C1=O	INACTIVE	5.96	10
S1CC[C@H](NC(=O)CCCCCC)C1=O	AGONIST	6.1	10
S1CC[C@H](NC(=O)CCCCCCC)C1=O	AGONIST	6.85	10
S1CC[C@H](NC(=O)Cc2ccccc2)C1=O	INACTIVE	5.6	10

S1CC[C@H](NC(=O)Cc2cc([N+](=O)[O-])ccc2)C1=O	ANTAGONIST	5.39	10
S1CC[C@H](NC(=O)Cc2ccc(OC)cc2)C1=O	INACTIVE	5.14	10
Brc1ccc(cc1)CC(=O)N[C@H]1CCOC1=O	ANTAGONIST	5.32	11
ClCC(=O)NCCCCCCCCC(=O)CC(=O)N[C@H]1CCOC1=O	ANTAGONIST	5.51	11
BrCC(=O)NCCCCCCCCC(=O)CC(=O)N[C@H]1CCOC1=O	INACTIVE	4.57	11
S=C=NCCCCCCCCC(=O)CC(=O)N[C@H]1CCOC1=O	INACTIVE	4.41	11
S=C=NCCCCCCCCCCC(=O)CC(=O)N[C@H]1CCOC1=O	ANTAGONIST	5.41	11
S=C=NCCCCCCCCCC(=O)CC(=O)N[C@H]1CCOC1=O	AGONIST	5.59	12
O(C)c1cc(NC(=O)CC(=O)CCCCCCCCC)ccc1	INACTIVE	3.7	12
Brc1cc(OCCCC(=O)N[C@H]2CCSC2=O)ccc1	INACTIVE	5.38	12
O=C(NC1CCCC1)CCCCCCCCC	INACTIVE	4.3	12
O=C(CC(=O)NCCCCCCCCC)c1ccccc1	ANTAGONIST	5.28	12
Clc1cc(ccc1)C(=O)Nc1ccccc1NC(=O)c1cc(Cl)ccc1	INACTIVE	4.16	12
O1CC[C@H](NC(=O)CC(=O)CCC)C1=O	INACTIVE	4.4	12
Oc1ccccc1NC(=O)CC(=O)CCCCCCCCC	INACTIVE	4.77	12
O1CC[C@H](NC(=O)CCCc2ccccc2)C1=O	INACTIVE	3.76	12
FC(F)(F)c1ccc(cc1)CCC(=O)N[C@H]1CCOC1=O	ANTAGONIST	5.52	12
Clc1ccc(OCCCC(=O)N[C@H]2CCOC2=O)cc1	INACTIVE	4.68	12
Brc1cc(Br)cc(CNC(=O)c2ccccc2Cl)c1OC(=O)c1ccc(F)cc1	ANTAGONIST	6.62	13
Brc1cc(Br)cc(CNC(=O)c2ccccc2Cl)c1OC(=O)c1ccccc1C	ANTAGONIST	7.27	13
Clc1cc(Cl)ccc1C(Oc1c(cc(cc1C)C)CNC(=O)c1ccccc1Cl)=O	ANTAGONIST	6.04	13
Brc1cc(Br)cc(CC(=O)Nc2ccccc2[N+](=O)[O-])c1OC(=O)CNC(=O)CCl	INACTIVE	4	14
Brc1cc(Br)cc(CC(=O)Nc2ccccc2[N+](=O)[O-])c1OC(=O)N1C(=O)C=CC1=O	INACTIVE	4.06	14
Brc1cc(Br)cc(CC(=O)Nc2ccccc2[N+](=O)[O-])c1OC(=O)CC	ANTAGONIST	5.44	14
Brc1cc(Br)cc(CC(=O)Nc2ccccc2[N+](=O)[O-])c1OC(=O)CCN1C(=O)C=CC1=O	INACTIVE	4.11	14
Brc1cc(Br)cc(CNC(=O)c2ccccc2[N+](=O)[O-])c1OC(=O)CN1C(=O)C=CC1=O	INACTIVE	4.28	14
Brc1cc(Br)cc(CNC(=O)c2ccccc2[N+](=O)[O-])c1OC(=O)CCN1C(=O)C=CC1=O	ANTAGONIST	5.82	14
O1CC[C@H](NC(=O)CC(=O)CCCCCCCCCC)C1=O	INACTIVE	4.52	14
[Br+](c1cc(CC(=O)Nc2ccccc2[N+](=O)[O-<br>])c(OC(=O)CCCN=C=S)c(Br)c1)[CH2-]	INACTIVE	3.83	14
[Br+](c1cc(CNC(=O)c2ccccc2[N+](=O)[O-<br>])c(OC(=O)CCCN=C=S)c(Br)c1)[CH2-]	INACTIVE	3.7	14
Brc1cc(Br)cc(CC(=O)Nc2ccccc2[N+](=O)[O-])c1OCCCN=C=S	INACTIVE	3.7	14
Brc1cc(Br)cc(CC(=O)Nc2ccccc2[N+](=O)[O-])c1OCCCCN=C=S	INACTIVE	3.84	14
Brc1cc(Br)cc(CC(=O)Nc2ccccc2[N+](=O)[O-])c1OC(=O)[N-]C(=O)CCl	INACTIVE	4.13	14
O1CC[C@H](NC(=O)CC(=O)CCCCCCCCC)C1=O	AGONIST	8.75	15
Brc1cc(Br)cc(CNC(=O)c2ccccc2[N+](=O)[O-])c1OC(=O)c1ccc(Br)cc1	AGONIST	8.79	15
Brc1cc(Br)cc(CNC(=O)c2ccccc2[N+](=O)[O-])c1OC(=O)c1ccc(OC)cc1	AGONIST	8.04	15
Brc1cc(Br)cc(CNC(=O)c2ccccc2[N+](=O)[O-])c1OC(=O)CCCCCCC	AGONIST	7.91	15
Brc1cc(Br)cc(CNC(=O)c2ccccc2[N+](=O)[O-])c1OC(=O)C	AGONIST	7.69	15
Brc1cc(Br)cc(CNC(=O)c2ccccc2[N+](=O)[O-])c1OCOC	AGONIST	7.42	15
Brc1cc(Br)cc(CNC(=O)c2ccccc2[N+](=O)[O-])c1OC	AGONIST	7.22	15
Brc1cc(Br)cc(CNC(=O)c2ccccc2[N+](=O)[O-])c1O	AGONIST	6.05	15
Brc1cc(Br)cc(CNC(=O)c2ccccc2Cl)c1OC(=O)c1ccccc1[N+](=O)[O-]	AGONIST	8.29	15
Brc1cc(Br)cc(CNC(=O)c2ccccc2Cl)c1OC(=O)c1ccccc1Cl	AGONIST	8.1	15
Fc1cc(ccc1)CNC(=O)c1ccccc1[N+](=O)[O-]	INACTIVE	4.68	16

FC(F)(F)c1ccc(cc1)CNC(=O)c1ccccc1[N+](=O)[O-]	INACTIVE	4.46	16
O=C(NC[C@@H](C)c1ccccc1)c1ccccc1[N+](=O)[O-]	INACTIVE	4.43	16
O=C(NCCc1ccccc1)c1ccccc1[N+](=O)[O-]	INACTIVE	4.68	16
O=C(NCCCCCC)c1ccccc1[N+](=O)[O-]	INACTIVE	4.4	16
O=C(NCCC=1CCCCC=1)c1ccccc1[N+](=O)[O-]	INACTIVE	4.28	16
O=C(NCc1cc([N+](=O)[O-])ccc1)c1ccccc1[N+](=O)[O-]	INACTIVE	4.6	16
O=C(NCCCCCCCCCCC)c1ccccc1[N+](=O)[O-]	AGONIST	6.45	16
Fc1cc(cc(F)c1)CNC(=O)c1ccccc1[N+](=O)[O-]	ANTAGONIST	5.18	16
Fc1cc(ccc1F)CNC(=O)c1ccccc1[N+](=O)[O-]	INACTIVE	4.8	16
Fc1cc(F)ccc1CNC(=O)c1ccccc1[N+](=O)[O-]	INACTIVE	4.35	16
Clc1cc(ccc1)CNC(=O)c1ccccc1[N+](=O)[O-]	ANTAGONIST	5.05	16
Brc1cc(ccc1)CNC(=O)c1ccccc1[N+](=O)[O-]	ANTAGONIST	5.02	16
Ic1cc(ccc1)CNC(=O)c1ccccc1[N+](=O)[O-]	ANTAGONIST	5.19	16
Brc1ccc(cc1)CCNC(=O)c1ccccc1[N+](=O)[O-]	ANTAGONIST	5.32	16
O=C(NCc1ccccc1[N+](=O)[O-])c1ccccc1[N+](=O)[O-]	ANTAGONIST	5.01	16
Brc1cc(Br)cc(CNC(=O)c2ccccc2[N+](=O)[O-])c1OC(=O)c1ccccc1	AGONIST	8.94	16
Brc1cc(Br)cc(CNC(=O)c2ccccc2[N+](=O)[O-])c1OC(=O)c1ccccc1[N+](=O)[O-]	AGONIST	9.17	16
Brc1cc(Br)cc(CNC(=O)c2ccccc2[N+](=O)[O-])c1OC(=O)c1ccccc1OC	AGONIST	8.96	16
Brc1cc(Br)cc(CNC(=O)c2ccccc2[N+](=O)[O-])c1OC(=O)c1ccccc1C#N	AGONIST	8.25	16
Brc1cc(Br)cc(CNC(=O)c2ccccc2[N+](=O)[O-])c1OC(=O)c1ccc(Cl)cc1	AGONIST	8.68	16
BrC/1=CC(O\C\1=C/Br)=O	ANTAGONIST	5.7	17
S(Cc1ccccc1)CC(=O)N[C@@H]1CCOC1=O	INACTIVE	4	17
S(CCc1ccccc1)CC(=O)N[C@@H]1CCOC1=O	INACTIVE	4	17
S(CC(=O)N[C@@H]1CCOC1=O)c1cc2c(cc1)cccc2	INACTIVE	3.82	17
S(CCCCCCC)CC(=O)NC1CCCC1	INACTIVE	3.82	17
S(CCCCCCC)CC(=O)NC1CCCCC1	INACTIVE	4.12	17
S(CCCCCCC)CC(=O)N[C@@H]1CCC[C@H]1O	INACTIVE	4	17
S(CCCCCCC)CC(=O)N[C@@H]1CCCC1=O	INACTIVE	3.82	17
S(CCCCCCC)CC(=O)N[C@@H]1CCCCC1=O	INACTIVE	3.82	17
S(CCCCC)CC(=O)N[C@@H]1CCOC1=O	INACTIVE	3.52	17
S(=O)(CCCCC)CC(=O)N[C@H]1CCOC1=O	INACTIVE	3.52	17
S(=O)(CCCCCCC)CC(=O)N[C@H]1CCOC1=O	INACTIVE	4.3	17
S(CCCCCCC)CC(=O)N[C@@H]1CCOC1=O	ANTAGONIST	5.22	17
S(CCCCCCCCC)CC(=O)N[C@@H]1CCOC1=O	INACTIVE	4.3	17
S(CC(=O)N[C@@H]1CCOC1=O)c1ccccc1	INACTIVE	3.52	17
S(CC(=O)N[C@@H]1CCOC1=O)c1ccc(cc1)C	INACTIVE	4.3	17
S(CC(=O)N[C@@H]1CCOC1=O)c1ccc(O)cc1	INACTIVE	4	17
S1CC[C@@H](NC(=O)Cn2c3c(nc2)N(C)C(=O)N(C)C3=O)C1=O	INACTIVE	3.7	18
O(CC(=O)NN1C(=Nc2c(cccc2)C1=O)C)c1ccccc1	INACTIVE	3.92	18
O=C(Nc1ncccc1C)c1n(nc(c1)C(=O)Nc1ncccc1C)C	INACTIVE	4.46	18
s1ccnc1NS(=O)(=O)c1ccc(NS(=O)(=O)c2ccccc2[N+](=O)[O-])cc1	INACTIVE	4.22	18
s1ccnc1NS(=O)(=O)c1ccc(N)cc1	ANTAGONIST	5.05	18
O1CC[C@H](NC(=O)Cn2nnc(c2)CCCC)C1=O	INACTIVE	4.75	19
O1CC[C@H](NC(=O)Cn2nnc(c2)CCCCC)C1=O	ANTAGONIST	5.49	19
O1CC[C@H](NC(=O)Cn2nnc(c2)C2CCCC2)C1=O	INACTIVE	4.79	19
O1CC[C@H](NC(=O)Cn2nnc(c2)C2CCCCC2)C1=O	INACTIVE	4.91	19

O1CC[C@H](NC(=O)Cn2nnc(c2)-c2ccccc2)C1=O	INACTIVE	4.8	19
Fc1cc(cc(F)c1)-c1nnn(c1)CC(=O)N[C@H]1CCOC1=O	INACTIVE	4.86	19
O1CC[C@H](NC(=O)CCc2nnn(c2)-c2ccc(OC)cc2)C1=O	INACTIVE	4.78	19
O1CC[C@H](NC(=O)CCc2nnn(c2)-c2ccc(cc2)C)C1=O	INACTIVE	4.96	19
O1CC[C@H](NC(=O)CCc2nnn(c2)-c2cc(N)ccc2)C1=O	ANTAGONIST	5.39	19
O=C1NC=Nc2[nH]nnc12	ANTAGONIST	6.14	20
Fc1cc(ccc1)C[C@@H]([NH3+])C(=O)[O-]	ANTAGONIST	5.74	20
O=C1NC(Nc2[nH]nnc12)=N	ANTAGONIST	6.19	20
O=C([O-])[C@@H]([NH3+])[C@H](O)c1ccccc1	ANTAGONIST	5.44	20
O=C([O-])c1c2c([nH]c1)cccc2	ANTAGONIST	5.68	20

References

1) Ahumedo, M., Díaz, A. & Vivas-Reyes, R. Theoretical and structural analysis of the active site of the transcriptional regulators LasR and TraR, using molecular docking methodology for identifying potential analogues of acyl homoserine lactones (AHLs) with anti-quorum sensing activity. Eur. J. Med. Chem. 45, 608–615 (2010).
2) Boursier, M. E., Manson, D. E., Combs, J. B. & Blackwell, H. E. A comparative study of non-native N-acyl l-homoserine lactone analogs in two Pseudomonas aeruginosa quorum sensing receptors that share a common native ligand yet inversely regulate virulence. Bioorg. Med. Chem. 26, 5336–5342 (2018).
3) Brackman, G. et al. Synthesis and evaluation of the quorum sensing inhibitory effect of substituted triazolyldihydrofuranones. Bioorg. Med. Chem. 20, 4737–4743 (2012).
4) Capilato, J. N. et al. Development of a novel series of non-natural triaryl agonists and antagonists of the Pseudomonas aeruginosa LasR quorum sensing receptor. Bioorg. Med. Chem. 25, 153–165 (2017).
5) Mendez, D. et al. ChEMBL: Towards direct deposition of bioassay data. Nucleic Acids Res. 47, D930–D940 (2019).
6) Gerdt, J. P., McInnis, C. E., Schell, T. L., Rossi, F. M. & Blackwell, H. E. Mutational Analysis of the Quorum-Sensing Receptor LasR Reveals Interactions that Govern Activation and Inhibition by Nonlactone Ligands. Chem. Biol. 21, 1361–1369 (2014).
7) Hansen, M. R. et al. Triazole-containing N-acyl homoserine lactones targeting the quorum sensing system in Pseudomonas aeruginosa. Bioorg. Med. Chem. 23, 1638–1650 (2015).
8) Lowery, C. A., Salzameda, N. T., Sawada, D., Kaufmann, G. F. & Janda, K. D. Medicinal Chemistry as a Conduit for the Modulation of Quorum Sensing. J. Med. Chem. 53, 7467–7489 (2010).
9) Malladi, V. L. A. et al. Substituted lactam and cyclic azahemiacetals modulate Pseudomonas aeruginosa quorum sensing. Bioorg. Med. Chem. 19, 5500–6 (2011).
10) McInnis, C. E. & Blackwell, H. E. Thiolactone modulators of quorum sensing revealed through library design and screening. Bioorg. Med. Chem. 19, 4820–4828 (2011).
11) Amara, N. et al. Fine-Tuning Covalent Inhibition of Bacterial Quorum Sensing. ChemBioChem 17, 825–835 (2016).
12) Welsh, M. A., Eibergen, N. R., Moore, J. D. & Blackwell, H. E. Small Molecule Disruption of Quorum Sensing Cross-Regulation in Pseudomonas aeruginosa Causes Major and Unexpected Alterations to Virulence Phenotypes. (2015).
13) Müh, U. et al. Novel Pseudomonas aeruginosa quorum-sensing inhibitors identified in an ultra-high-throughput screen. Antimicrob. Agents Chemother. 50, 3674–9 (2006).
14) O'Brien, K. T., Noto, J. G., Nichols-O'Neill, L. & Perez, L. J. Potent Irreversible Inhibitors of LasR Quorum Sensing in Pseudomonas aeruginosa. ACS Med. Chem. Lett. 6, 162–167 (2015).
15) O'Reilly, M. C. et al. Structural and Biochemical Studies of Non-native Agonists of the LasR Quorum-Sensing Receptor Reveal an L3 Loop "Out" Conformation for LasR. Cell Chem. Biol. 25, 1128-1139.e3 (2018).

16) O'Reilly, M. C. & Blackwell, H. E. Structure-Based Design and Biological Evaluation of Triphenyl Scaffold-Based Hybrid Compounds as Hydrolytically Stable Modulators of a LuxR-Type Quorum Sensing Receptor. ACS Infect. Dis. 2, 32–38 (2016).
17) Pesci, E. C., Pearson, J. P., Seed, P. C. & Iglewski, B. H. Regulation of las and rhl quorum sensing in Pseudomonas aeruginosa. J. Bacteriol. 179, 3127–32 (1997).
18) Skovstrup, S. et al. Identification of LasR Ligands through a Virtual Screening Approach. ChemMedChem 8, 157–163 (2013).
19) Stacy, D. M. et al. Synthesis and biological evaluation of triazole-containing N-acyl homoserine lactones as quorum sensing modulators. Org. Biomol. Chem. 11, 938–954 (2013).
20) Yang, S. et al. Bicyclic brominated furanones: a new class of quorum sensing modulators that inhibit bacterial biofilm formation. Bioorg. Med. Chem. 22, 1313–7 (2014).

b. PqsR dataset

Molecule	Activity	pIC_{50}	Reference
O=C(N)c1ccc(cc1)C(C)(C)C	AGONIST	6.05	1
o1c(nnc1N)-c1cc(ccc1)C	INACTIVE	4.91	1
FC(F)(F)c1cc(ccc1)-c1oc(nn1)C	INACTIVE	4.89	1
FC(F)(F)c1cc(ccc1)-c1ocnn1	INACTIVE	5.19	1
s1cccc1-c1[nH]nc(c1)C(F)(F)F	INACTIVE	5.07	1
o1c(nnc1N)-c1cc(ccc1)C(C)(C)C	INACTIVE	5.51	1
O=C(N)c1cc2c(cc1)cccc2	AGONIST	6.1	1
Brc1cc(ccc1)C(=O)N	INACTIVE	5.17	1
Brc1nc(N)ccc1	INACTIVE	5.17	1
Clc1ccc(cc1)-c1oc(nn1)N	INACTIVE	3.7	1
Clc1ccc(cc1)-c1sc(nn1)N	INACTIVE	5.43	1
o1c(nnc1N)-c1cc(ccc1)C#N	INACTIVE	4.75	1
FC(F)(F)c1cc(ccc1)-c1oc(nn1)N	AGONIST	5.89	1
Clc1cc(ccc1)-c1oc(nn1)N	INACTIVE	4.57	1
Fc1cc2N=C(N([O-])C(=O)c2cc1)CCCCCCCCC	INACTIVE	4.68	2
Clc1cc2N=C(N(OC)C(=O)c2cc1)CCCCCCCCC	INACTIVE	4.43	2
Fc1cc2N=C(N(OC)C(=O)c2cc1)CCCCCCCCC	AGONIST	5.66	2
Fc1cc2c(N=C(N(OC)C2=O)CCCCCCCCC)cc1F	INACTIVE	4.27	2
O=C1N(N)C(=Nc2c1cccc2)CCCCCCC	INACTIVE	4.27	2
O=C1N(N)C(=Nc2c1cccc2)CCCCCCCCC	INACTIVE	4.11	2
O=C1N(N)C(=Nc2c1cccc2)CCCCCCCCCCC	INACTIVE	3.68	2
Clc1cc2N=C(N(N)C(=O)c2cc1)CCCCCCCCC	ANTAGONIST	5.3	2
Fc1cc2N=C(N(N)C(=O)c2cc1)CCCCCCCCC	ANTAGONIST	5.41	2
Fc1cc2c(N=C(N(N)C2=O)CCCCCCCCC)cc1F	ANTAGONIST	5.92	2
O=C1N(CC[NH3+])C(=Nc2c1cccc2)CCCCCCCCC	INACTIVE	3.77	2
Clc1cc2c(N=C(N(CC[NH3+])C2=O)CCCCCCCCC)cc1	ANTAGONIST	5.03	2
Clc1cc2N=C(N(CC[NH3+])C(=O)c2cc1)CCCCCCCCC	INACTIVE	4.71	2
Clc1cc2N=C(N(CCC[NH3+])C(=O)c2cc1)CCCCCCCCC	INACTIVE	4.81	2
Clc1cc2N=C(N(CC[NH3+])C(=O)c2cc1)CCCc1ccccc1	INACTIVE	4.1	2
Clc1cc2N=C(N(N)C(=O)c2cc1)CCCc1ccccc1	INACTIVE	4.4	2
O=C1C=C(Nc2c1cccc2)CCCCCCCCC	AGONIST	6.4	2

OC=1C(=O)c2c(NC=1CCCCCCCCC)cccc2	AGONIST	5.96	2
Clc1cc2NC(CCCCCCC)=C(O)C(=O)c2cc1	AGONIST	7.85	2
O=C1c2c(NC(CCCCCCC)=C1N)cccc2	AGONIST	6.4	2
O=C1N([O-])C(=Nc2c1cccc2)CCCCCCC	INACTIVE	4.27	2
O=C1N([O-])C(=Nc2c1cccc2)CCCCCCCCC	INACTIVE	4.52	2
Clc1cc2N=C(N([O-])C(=O)c2cc1)CCCCCCCCC	INACTIVE	4.9	2
FC(F)(F)c1cc2c(NC(=CC2=O)CCCCCCC)cc1	ANTAGONIST	7.27	3
O=C1C=C(Nc2c1cc([N+](=O)[O-])cc2)CCCCOCC	ANTAGONIST	5.45	3
O=C1c2cc([N+](=O)[O-])ccc2NC(CCCCCCC)=C1CO	ANTAGONIST	7.14	3
O=C1c2cc([N+](=O)[O-<br>])ccc2NC(CCCCCCC)=C1C(=O)N	ANTAGONIST	7.46	3
O=C1C=C(Nc2c1cc([N+](=O)[O-])cc2)CCCCCCC	ANTAGONIST	7.29	3
OC=1C(=O)c2c(NC=1CCCCCCC)cccc2	AGONIST	8.2	3
OC=1C(=O)c2cc([N+](=O)[O-])ccc2NC=1CCCCCCC	AGONIST	8.55	3
FC(F)(F)c1cc2c(N(C)C(=CC2=O)CCCCCCC)cc1	ANTAGONIST	6.57	3
FC(F)(F)c1cc2c(N(C)C(=CC2=O)CCCCCC)cc1	ANTAGONIST	6.64	3
FC(F)(F)c1cc2c(NC(=CC2=O)COCCCCC)cc1	ANTAGONIST	6.76	3
O=C1C=C(Nc2c1cc([N+](=O)[O-])cc2)COCCCCC	ANTAGONIST	6.6	3
FC(F)(F)c1cc2c(NC(=CC2=O)CCCCOCC)cc1	ANTAGONIST	5.77	3
O=C(N[O-])c1ccc(cc1)C(C)(C)C	INACTIVE	4.63	4
S(=O)(=O)(C)c1nc(-n2nnnc2)ccn1	INACTIVE	4.82	4
S(=O)(=O)(CCCCCC)c1nc(-n2nncc2)ccn1	INACTIVE	4.3	4
Brc1cc(ccc1)-c1oc(nn1)N	ANTAGONIST	5.12	5
FC(F)(F)Oc1cc(ccc1)-c1oc(nn1)N	INACTIVE	4.33	5

References

1) Mendez, D. et al. ChEMBL: Towards direct deposition of bioassay data. Nucleic Acids Res. 47, D930–D940 (2019).
2) Ilangovan, A. et al. Structural Basis for Native Agonist and Synthetic Inhibitor Recognition by the Pseudomonas aeruginosa Quorum Sensing Regulator PqsR (MvfR). PLoS Pathog. (2013).
3) Lu, C. et al. Discovery of Antagonists of PqsR, a Key Player in 2-Alkyl-4-quinolone-Dependent Quorum Sensing in Pseudomonas aeruginosa. Chem. Biol. 19, 381–390 (2012).
4) Soheili, V., Tajani, A. S., Ghodsi, R. & Bazzaz, B. S. F. Anti-PqsR compounds as next-generation antibacterial agents against Pseudomonas aeruginosa: A review. Eur. J. Med. Chem. 172, 26–35 (2019).
5) Zender, M. et al. Discovery and Biophysical Characterization of 2-Amino-oxadiazoles as Novel Antagonists of PqsR, an Important Regulator of Pseudomonas aeruginosa Virulence. J. Med. Chem. 56, 6761–6774 (2013).

c. RhlR dataset

Molecule	Activity	pIC_{50}	Reference
O1CC[C@H](NC(=O)CCC)C1=O	INACTIVE	5.09	1
O1CC[C@H](NC(=O)CC2CC2)C1=O	AGONIST	5.56	1
S1CC[C@H](NC(=O)CCC)C1=O	AGONIST	5.42	1
O=C1CCC[C@@H]1NC(=O)CCC	INACTIVE	4.84	1
O1CC[C@H](NC(=O)CCC=C)C1=O	INACTIVE	5.1	1
O=C1CCC[C@@H]1NC(=O)C1CCC1	INACTIVE	5.23	1

Ic1ccc(OCC(=O)NC[C@H]2OCCC2)cc1	INACTIVE	4	1
Ic1ccc(OCC(=O)N[C@H]2CCSC2=O)cc1	INACTIVE	4.5	1
S1CC[C@H](NC(=O)C2CCC2)C1=O	AGONIST	5.76	1
O=C1CCC[C@@H]1NC(=O)CC(C)C	INACTIVE	5.12	1
S1CC[C@H](NC(=O)CC(C)C)C1=O	AGONIST	6.33	1
O1CC[C@H](NC(=O)CC(C)C)C1=O	AGONIST	5.99	1
O=C(NC1CCCC1)C1CCC1	INACTIVE	4.57	1
O1CC[C@H](NC(=O)C2CCCC2)C1=O	AGONIST	5.91	1
O1CC[C@H](NC(=O)CCCC)C1=O	INACTIVE	4.97	1
O1CC[C@H](NC(=O)C(C)C)C1=O	AGONIST	5.31	1
O1CC[C@H](NC(=O)[C@@H](CC)C)C1=O	INACTIVE	5.11	1
O1CC[C@H](NC(=O)C\C=C\C)C1=O	INACTIVE	5.16	1
O1CC[C@H](NC(=O)C2CCC2)C1=O	AGONIST	5.85	1
O1CC[C@H](NC(=O)Cc2cc(ccc2)C)C1=O	AGONIST	5.7	2
O1CC[C@H](NC(=O)Cc2cc(OC)ccc2)C1=O	AGONIST	5.33	2
O1CC[C@H](NC(=O)Cc2cc(ccc2)C#N)C1=O	AGONIST	5.77	2
S(C)c1cc(ccc1)CC(=O)N[C@H]1CCOC1=O	INACTIVE	5.18	2
Clc1ccc(cc1)CCC(=O)N[C@H]1CCOC1=O	INACTIVE	5.18	2
Brc1cc(ccc1)CCC(=O)N[C@H]1CCOC1=O	INACTIVE	4.95	2
O1CC[C@H](NC(=O)CCc2ccc([N+](=O)[O-])cc2)C1=O	INACTIVE	4.57	2
O1CC[C@H](NC(=O)CC2CCCC2)C1=O	AGONIST	5.51	2
Ic1ccc(cc1)CC(=O)N[C@H]1CCOC1=O	ANTAGONIST	5.09	2
O1CC[C@H](NC(=O)Cc2ccc([N+](=O)[O-])cc2)C1=O	INACTIVE	4.75	2
O1CC[C@H](NC(=O)Cc2ccc(cc2)C)C1=O	INACTIVE	4.7	2
FC(F)(F)c1ccc(cc1)CC(=O)N[C@H]1CCOC1=O	INACTIVE	4.61	2
Clc1cc(ccc1Cl)CC(=O)N[C@H]1CCOC1=O	ANTAGONIST	5.47	2
O1CC[C@H](NC(=O)Cc2ccccc2)C1=O	INACTIVE	4.83	2
O1CC[C@H](NC(=O)Cc2cc3c(cc2)cccc3)C1=O	INACTIVE	4.68	2
O1CC[C@H](NC(=O)COc2ccc(cc2)C)C1=O	ANTAGONIST	4.97	2
O1CC[C@H](NC(=O)COc2ccc(OC)cc2)C1=O	ANTAGONIST	4.92	2
Brc1ccc(OCC(=O)N[C@H]2CCOC2=O)cc1	ANTAGONIST	5.23	2
Ic1ccc(OCC(=O)N[C@H]2CCOC2=O)cc1	INACTIVE	4.76	2
S(C)c1ccc(cc1)CCC(=O)N[C@H]1CCOC1=O	INACTIVE	4.66	2
Fc1ccc(cc1)CC(=O)N[C@H]1CCOC1=O	INACTIVE	5.05	2
Clc1cc(ccc1)CC(=O)N[C@H]1CCOC1=O	INACTIVE	5.26	2
Ic1cc(ccc1)CC(=O)N[C@H]1CCOC1=O	INACTIVE	5.24	2
Brc1cc(OCCCC(=O)N[C@H]2CCSC2=O)ccc1	ANTAGONIST	5.4	3
[Br+](c1cc(OCCCC(=O)N[C@@H]2CCSC2=O)ccc1)[CH2-]	INACTIVE	4	3
Clc1cc(OCCCC(=O)N[C@@H]2CCSC2=O)ccc1	ANTAGONIST	5.05	3
S=C=NCCCCCCCCCC(=O)CC(=O)N[C@H]1CCOC1=O	INACTIVE	4.25	3
O=C(CC(=O)NCCCCCCCCC)c1ccccc1	INACTIVE	4.74	3

References

1) Blackwell, H. E., Boursier, M. E. & Moore, J. Synthetic ligands that modulate the activity of the rhlr quorum sensing receptor. (2017).
2) Eibergen, N. R., Moore, J. D., Mattmann, M. E. & Blackwell, H. E. Potent and Selective Modulation of the RhlR Quorum Sensing Receptor by Using Non-native Ligands: An Emerging Target for Virulence Control in Pseudomonas aeruginosa. ChemBioChem 16, 2348–2356 (2015).
3) O'Loughlin, C. T. et al. A quorum-sensing inhibitor blocks Pseudomonas aeruginosa virulence and biofilm formation. Proc. Natl. Acad. Sci. 110, 17981–17986 (2013).

2. Complexity graphs

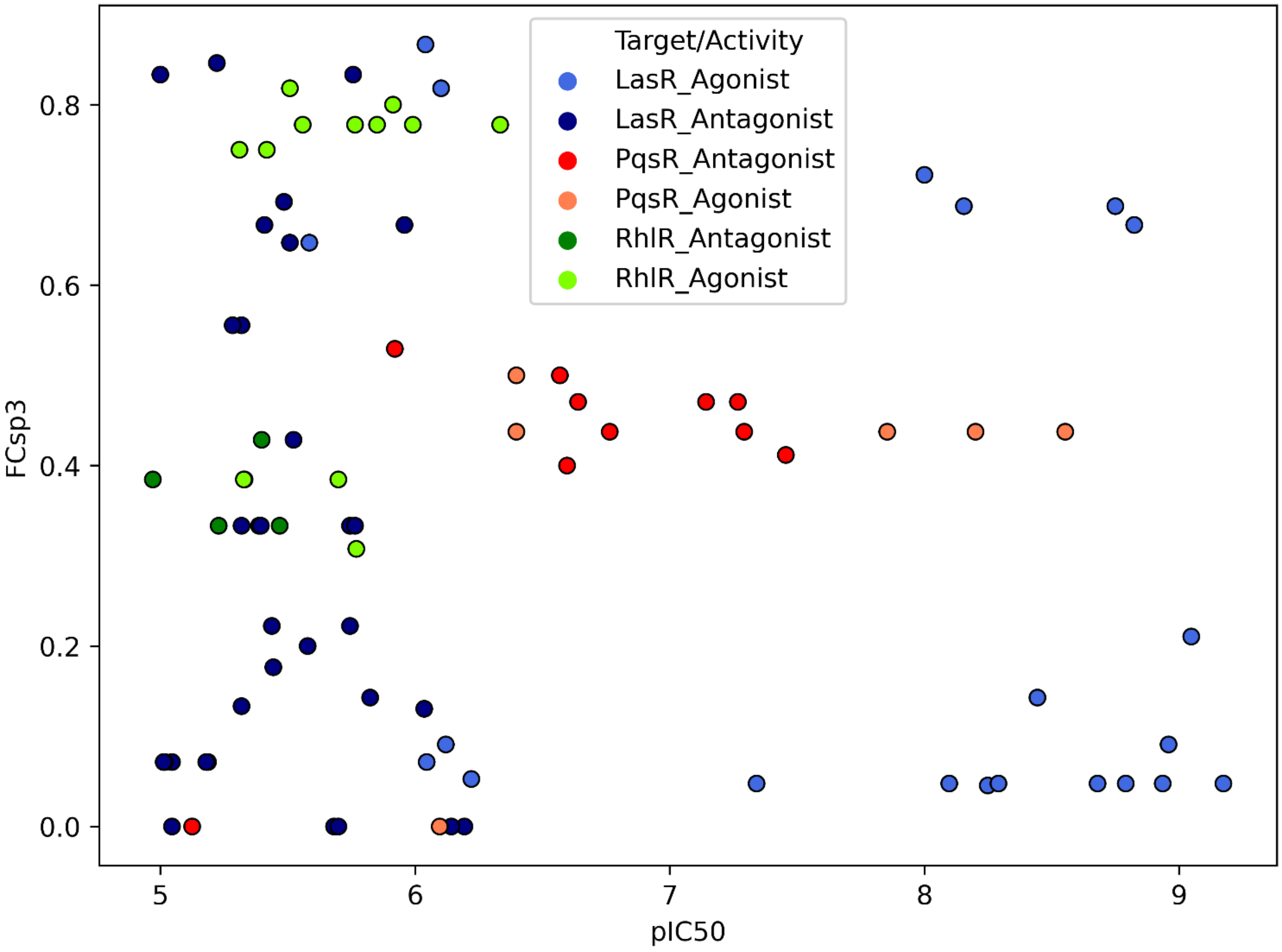

Fig. 1 Distribution of biological activity values against fraction of sp^3 carbons complexity. Molecules with activity against LasR, PqsR and RhlR appear in blue, red and green respectively; light colors represent agonist compounds and dark colors antagonist compounds.

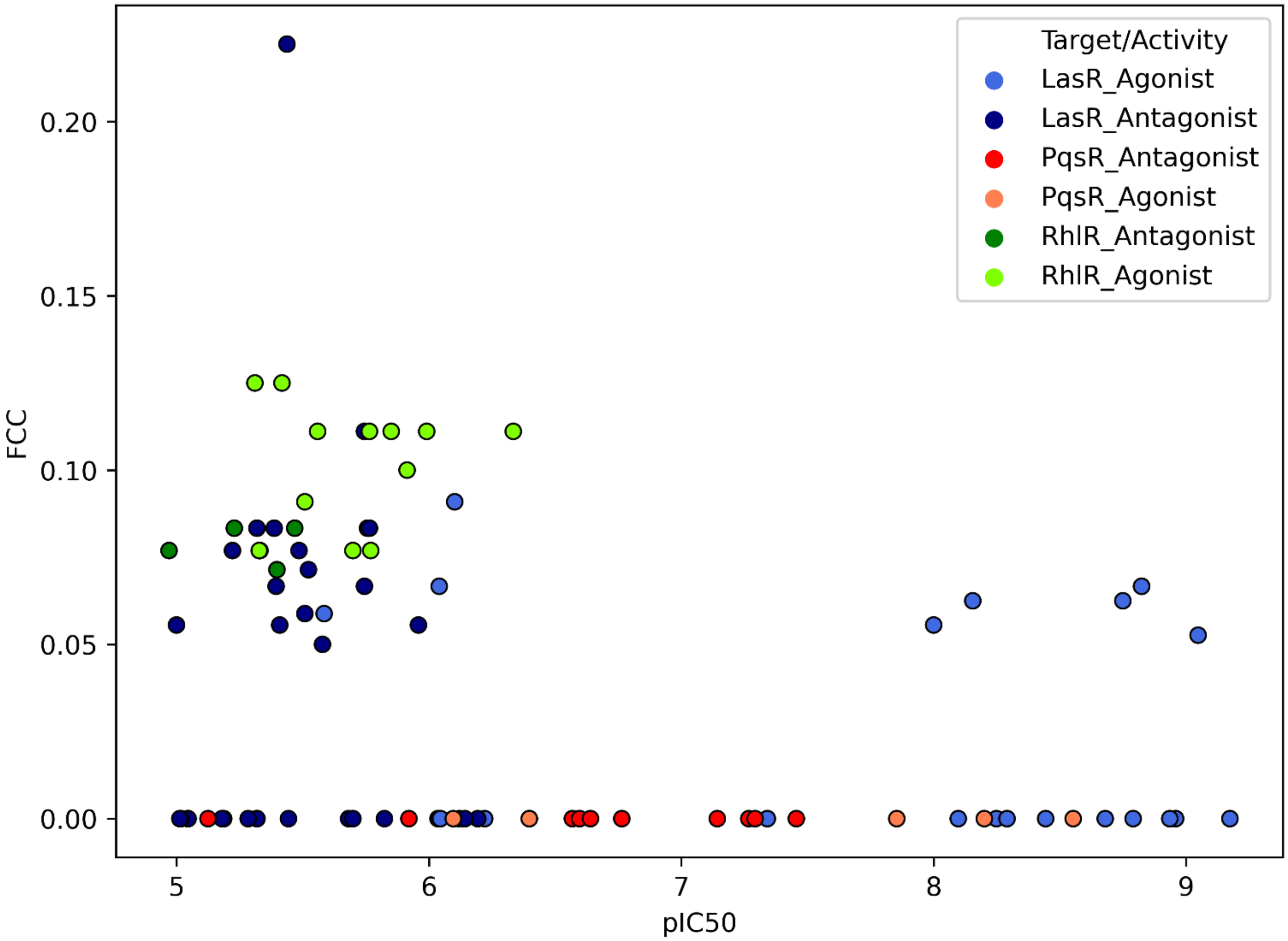

Fig. 2 Distribution of biological activity values against fraction of chiral centers complexity. Molecules with activity against LasR, PqsR and RhlR appear in blue, red and green respectively; light colours represent agonist compounds and dark colours antagonist compounds.

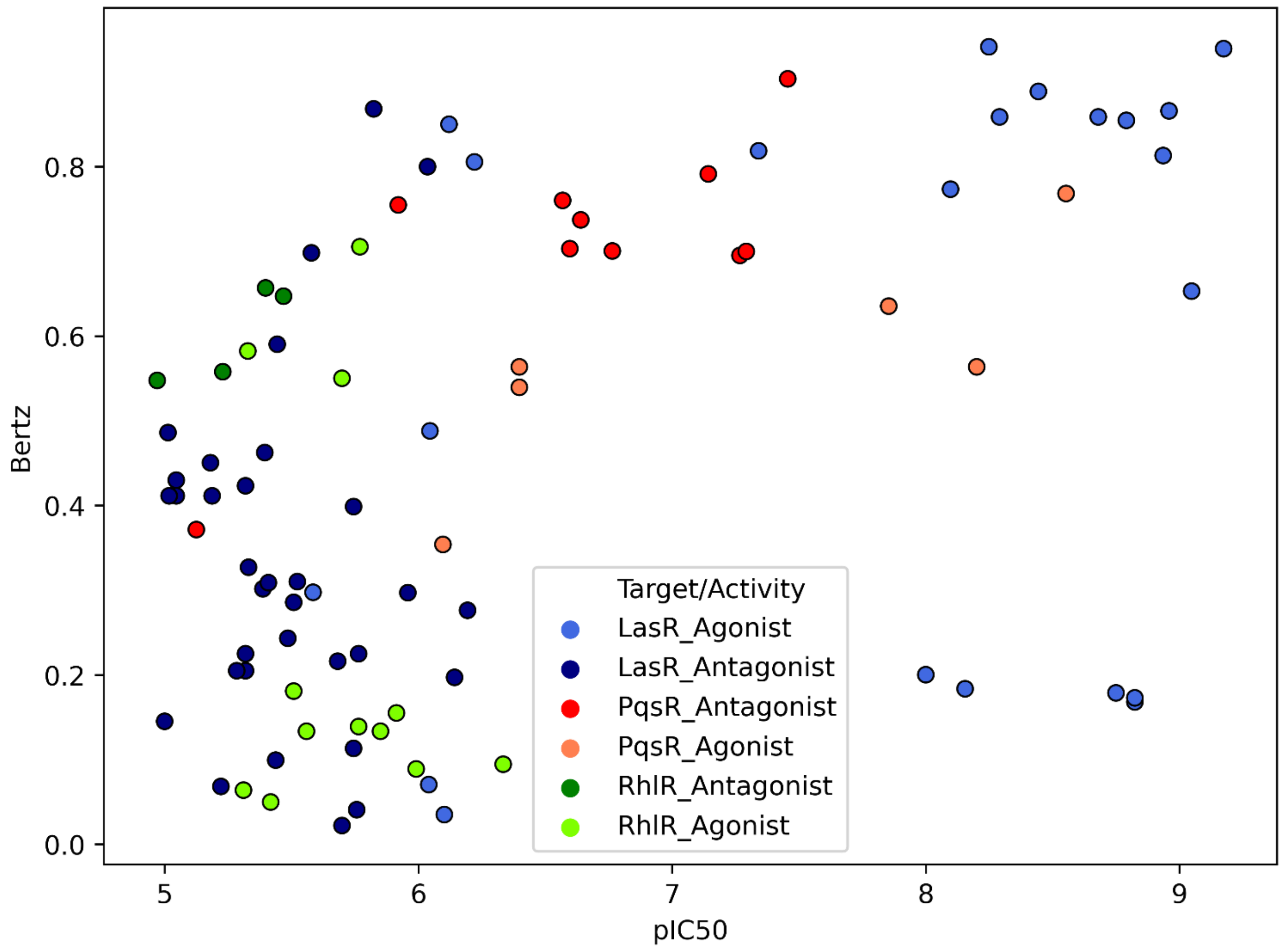

Fig. 3 Distribution of biological activity values against Bertz complexity. Molecules with activity against LasR, PqsR and RhlR appear in blue, red and green respectively; light colours represent agonist compounds and dark colours antagonist compounds.

3. Scaffolds founded

(a) LasR Scaffolds

Scafold	Frecuency
c1ccoc1	22
O=C(Cc1ccccc1OC(=O)c1ccccc1)Nc1ccccc1	21
O=C(NCc1ccccc1)c1ccccc1	16
O=C(NCc1ccccc1OC(=O)c1ccccc1)c1ccccc1	14
O=C1CCCO1	11
c1ccccc1	9
O=C(Cc1ccccc1)Nc1ccccc1	5
C1CCCC1	5
O=C(Cc1ccccc1)NC1CCSC1=O	4
O=C1CCCS1	4
O=C(CSc1ccccc1)NC1CCOC1=O	3
O=C(CCc1cn(-c2ccccc2)nn1)NC1CCOC1=O	3
O=C(NCCc1ccccc1)c1ccccc1	3
c1ccsc1	3
O=C(Cc1ccccc1)NC1CCOC1=O	3
O=C(Cn1ccnn1)NC1CCOC1=O	2
c1nn[nH]n1	2
O=C(CCc1ccccc1)NC1CCOC1=O	2
O=C(Cn1cc(-c2ccccc2)nn1)NC1CCOC1=O	2
O=C1CCCCC1	2
C1CCOC1	2
O=C1CCCC1	2
O=C(CCCn1cccc1)Oc1ccccc1CNC(=O)c1ccccc1	1
O=C(Cc1ccccc1OC(=O)c1ccccc1)N=c1cc[nH]cc1	1
C=C1C=CC(=O)O1	1
O=C(Cc1ccccc1OC(=O)CCn1cccc1)Nc1ccccc1	1
O=C(Cc1ccccc1OC(=O)CCN1C(=O)C=CC1=O)NC1CCOC1=O	1
O=C(COc1ccccc1)Nn1cnc2ccccc2c1=O	1
O=S(=O)(N=c1[nH]ccs1)c1ccccc1	1
O=S(=O)(N=c1[nH]ccs1)c1ccc(NS(=O)(=O)c2ccccc2)cc1	1
O=C(Cc1ccccc1OC(=O)c1ccccc1)N=c1cc[nH]cn1	1
O=C(CSCCc1ccccc1)NC1CCOC1=O	1
O=C(N=c1cccc[nH]1)c1cc(C(=O)N=c2cccc[nH]2)[nH]n1	1
O=C(Oc1ccccc1CNC(=O)c1ccncn1)c1ccccc1	1
O=C(NCCC1=CCCCC1)c1ccccc1	1
O=C([CH-]c1ccccc1)Nc1ccccc1	1
O=C(Cc1ccccc1OC(=O)c1ccccc1)NC1CCOC1=O	1

c1ccc2[nH]ccc2c1	1
O=C1OCCC1n1ccnn1	1
O=C(Cn1cnc2[nH]c(=O)[nH]c(=O)c21)NC1CCSC1=O	1
O=C(Cc1ccccc1OC(=O)CCN1C(=O)C=CC1=O)Nc1ccccc1	1
O=C(Cn1cc(C2CCCC2)nn1)NC1CCOC1=O	1
O=C(CCCOc1ccccc1)NC1CCSC1=O	1
O=C(CSc1ccc2ccccc2c1)NC1CCOC1=O	1
O=C(CCCc1ccccc1)NC1CCOC1=O	1
O=C(CSCc1ccccc1)NC1CCOC1=O	1
O=c1[nH]cnc2[nH]nnc12	1
O=C(Cc1ccccc1OC(=O)c1ccccc1)N=c1cccc[nH]1	1
O=C(Cn1cc(C2CCCCC2)nn1)NC1CCOC1=O	1
O=C(CCCCCCCCCC1CO1)Nc1ccoc1	1
O=C(CCCOc1ccccc1)NC1CCOC1=O	1
O=C(Cc1ccc(-c2ccccc2)cc1)NC1CCSC1=O	1
O=C(Oc1ccccc1CNC(=O)c1ccccn1)c1ccccc1	1
O=C(CCN1C(=O)C=CC1=O)Oc1ccccc1CNC(=O)c1ccccc1	1
N=c1[nH]c(=O)c2[nH]nnc2[nH]1	1
O=C(NCc1ccccc1OC(=O)c1ccccc1)c1cccnc1	1
O=C(Cc1ccccc1OCc1ccccc1)Nc1ccccc1	1
C1CCCCC1	1
O=C(Cc1ccccc1OC(=O)N1C(=O)C=CC1=O)Nc1ccccc1	1
O=C(NC1CCOC1=O)c1ccc(-n2cc(CSc3ccccc3)nn2)cc1	1
O=C(Nc1ccccc1NC(=O)c1ccccc1)c1ccccc1	1
c1cc[nH]c1	1
O=C(CCCc1c[nH]c2ccccc12)NC1CCOC1=O	1
O=C(COc1ccccc1)NC1CCOC1=O	1
O=C(CN1C(=O)C=CC1=O)Oc1ccccc1CNC(=O)c1ccccc1	1
O=C(CCc1cc2ccccc2[nH]1)NC1CCSC1=O	1
O=C(CCCc1ccccc1)Nc1ccoc1	1
O=C(CCCN1C(=O)C=CC1=O)Oc1ccccc1CNC(=O)c1ccccn1	1
O=C(CC1C=CCC1)NC1CCOC1=O	1
O=C(Cc1ccccc1OC(=O)CCCN1C(=O)C=CC1=O)NC1CCOC1=O	1

(b) PqsR Scaffolds

Scaffold	Frecuency
O=c1[nH]cnc2ccccc12	17
O=c1cc[nH]c2ccccc12	16
N=c1[nH]nc(-c2ccccc2)o1	8
c1ccccc1	3
c1ccc(-c2nnco2)cc1	2

O=c1[nH]c(CCCc2ccccc2)nc2ccccc12	2
N=c1cccc[nH]1	1
N=c1[nH]nc(-c2ccccc2)s1	1
c1ccc2ccccc2c1	1
c1cc(-n2cnnn2)ncn1	1
c1csc(-c2ccn[nH]2)c1	1
c1cc(-n2ccnn2)ncn1	1

(c) RhlR Scaffolds

Scaffold	Frecuency
O=C(Cc1ccccc1)NC1CCOC1=O	13
O=C1CCCO1	6
O=C(CCc1ccccc1)NC1CCOC1=O	4
O=C(COc1ccccc1)NC1CCOC1=O	4
O=C1CCCS1	2
O=C1CCCC1	2
O=C(CCCOc1ccccc1)NC1CCSC1=O	2
c1ccoc1	2
O=C(COc1ccccc1)NCC1CCCO1	1
c1ccccc1	1
O=C(NC1CCCC1)C1CCC1	1
O=C(NC1CCSC1=O)C1CCC1	1
O=C([CH-]CCOc1ccccc1)NC1CCSC1=O	1
O=C(Cc1ccc2ccccc2c1)NC1CCOC1=O	1
O=C(NC1CCCC1=O)C1CCC1	1
O=C(CC1CC1)NC1CCOC1=O	1
O=C(NC1CCOC1=O)C1CCCC1	1
O=C(NC1CCOC1=O)C1CCC1	1
O=C(CC1CCCC1)NC1CCOC1=O	1
O=C(COc1ccccc1)NC1CCSC1=O	1
